# Effect of a diuretic adjustment algorithm and nonpharmacologic management in patients with heart failure: study protocol for a randomized controlled trial

**DOI:** 10.1186/s13063-015-0559-7

**Published:** 2015-02-08

**Authors:** Maria Karolina Echer Ferreira Feijó, Andreia Biolo, Karen Brasil Ruschel, Letícia Orlandin, Graziella Badin Aliti, Eneida Rejane Rabelo-Silva

**Affiliations:** School of Nursing, Graduate Program Federal University of Rio Grande do Sul, São Manoel, 963 - Rio Branco, Porto Alegre, RS 90620-110 Brazil; Heart Failure and Transplant Group, Cardiovascular Division, Hospital de Clínicas de Porto Alegre, Porto Alegre, Brazil; Graduate Program in Health Sciences: Cardiology and Cardiovascular Sciences, Federal University of Rio Grande do Sul, Porto Alegre, Brazil

**Keywords:** Heart failure, Algorithms, Randomized controlled trial, Diuretics, Patient readmission clinical evolution

## Abstract

**Background:**

One of the challenges in treating patients with heart failure (HF) is achieving clinical stability and reducing the hospital readmission rate. A diuretic dose adjustment algorithm developed in the United States (Diuretic Treatment Algorithm, DTA) and later validated for use in Brazil (as the Algoritmo de Ajuste de Diurético, AAD) has proved feasible and readily applicable, but its effect on clinical outcomes has yet to be assessed. This report aims to describe a randomized clinical trial protocol designed to assess the effectiveness of the AAD and of nonpharmacologic management in improving clinical stability and reducing the readmission rate at 90 days in patients with HF.

**Methods/Design:**

A PROBE (prospective randomized open blinded endpoint) parallel-group design will be used. Adult patients with a diagnosis of reduced ejection fraction HF, who are being treated at a specialized HF clinic are being recruited. Those with indications for loop diuretic dose adjustment during routine clinic visits will be randomized to take part in the trial. Participants in the intervention group (IG) shall have their diuretic doses adjusted in accordance with the AAD and receive four telephone calls (one per week) over 30 days to reinforce guidance on nonpharmacological management (fluid and sodium restriction). Participants in the control group (CG) shall have their diuretic doses adjusted by a physician during the first trial visit and shall not receive any telephone calls. Patients in both groups shall return at 1 month for face-to-face reassessment. The study endpoints shall comprise readmission and/or emergency department visits due to HF decompensation within 90 days and clinical instability. All participants shall be required to have a scale at home (or easy access to one), a telephone number, agree to telephone-based follow-up, and be available to return for a 1-month trial visit. Overall, 135 patients are expected to be enrolled in each group.

**Discussion:**

This trial shall assess the effectiveness of the AAD algorithm and non-pharmacologic management by early identification of clinical deterioration and establishment of a combined intervention to reduce emergency department visits, readmission rate, or a composite endpoint thereof.

**Trial registration number:**

ClinicalTrials.gov Identifier, NCT02068937 (23 February 2014).

## Background

Heart failure (HF) is a highly prevalent clinical syndrome. In the United States alone, more than 5 million people have HF and more than 550,000 new cases are diagnosed each year. Complex issues involving physiological, psychological, social, and medical care make management of HF a challenge [1,2]. Over the last years, the annual number of hospitalizations with HF as a primary cause increased from 800,000 to over 1 million, and that of hospitalizations with HF as a secondary diagnosis, from 2.4 million to 3.6 million [3]. Approximately 50% of these patients are readmitted to hospital within 6 months of discharge, and 70% of these readmissions are associated with deterioration of HF [4,5]. The prevention of HF-related readmission has been the focus of several studies, and efforts are being made to reduce this rate to the 20% range [6].

There is a consensus in the literature that HF-related readmissions are prompted mainly by clinical decompensation due to congestive processes. The main manifestations of this decompensation are dyspnea, fatigue, lower extremity edema, paroxysmal nocturnal dyspnea, orthopnea, and jugular venous distension [7]. These signs and symptoms of decompensation are readily recognizable in a variety of settings, which enables rapid intervention by healthcare professionals. Several different care processes have the potential to reduce HF-related readmission rates, including comprehensive educational intervention during hospital admissions, medication reconciliation, support from the nursing team after hospital discharge, disease management, and improvement of communication between patients and providers [8]. One strategy that has been used as an adjuvant to conventional treatment is telemonitoring. This strategy, which has proven cost-effectiveness and can be implemented in different settings [9], enables early identification of clinical deterioration and prompt intervention, and has been demonstrated to reduce the risk of hospitalization for decompensated HF and reduce all-cause mortality [10-12].

Within this context, United States (U.S.) researchers developed an algorithm for over-the-phone adjustment of diuretic doses, with a focus on pharmacological and nonpharmacological assessment and home-based management of patients with HF. The Diuretic Treatment Algorithm (DTA) was demonstrated to be effective in preventing HF decompensation, significantly reducing 30-day readmission rates and decreasing HF-related hospitalizations by 50% [13]. The DTA was later translated and adapted for use in Brazil as the Algoritmo de Ajuste de Diurético (Diuretic Adjustment Algorithm, AAD) and validated in an outpatient sample [14]. The results of that study demonstrated the feasibility and applicability of the AAD in the Brazilian healthcare reality. However, the effects of use of this algorithm on clinical outcomes have yet to be tested.

The present report aims to describe a randomized clinical trial (RCT) protocol designed to assess the effectiveness of the AAD and of nonpharmacologic management in improving clinical stability and reducing the 90-day readmission rate in patients with HF.

## Methods/Design

### Study design

A PROBE (prospective randomized open blinded endpoint) parallel-group design shall be employed, with patients recruited from the specialty HF clinic of Hospital de Clínicas de Porto Alegre (HCPA), a university-affiliated hospital in Porto Alegre, Rio Grande do Sul, Brazil. Clinical assessment shall take place at the HCPA Clinical Research Center.

### Inclusion and exclusion criteria

Adult patients with a diagnosis of reduced ejection fraction HF who are being treated at a specialized HF clinic are being selected. The diagnosis of HF is established by clinical history (signs and symptoms), echocardiographic findings (left ventricular ejection fraction <45%) [15] and medical records confirming management at the HF clinic. Patients are required to have a medical indication for adjustment of loop diuretic (furosemide) dose during a clinic visit, have a scale at home or ready access to one, have a telephone number for follow-up contact, agree to receive such follow-up and be available to return to the hospital for a 1-month reassessment visit. The exclusion criteria are communication barriers or physical impairments that preclude body weight monitoring, degenerative neurological conditions, waitlist status for cardiac transplantation, scheduled surgery and chronic kidney disease requiring renal replacement therapy.

### Ethical considerations

All participants will read and sign an informed consent form before entering the study. The study protocol was approved by the HCPA Institutional Review Board (protocol number 10-0376) and will be conducted in accordance with the principles of the Declaration of Helsinki and in compliance with the Brazilian legal and regulatory framework for research involving human subjects.

### Heart Failure Clinic and team approach

The trial HF clinic is staffed by a multidisciplinary team of cardiologists, nurses, and dietitians. On average, 200 patients per month are seen at the clinic, with a focus on promoting adherence to optimized pharmacological and nonpharmacological management. During each visit, patients undergo a clinical assessment that includes use of the Clinical Congestion Score (CCS) [16]. All interventions seek to improve the health education process and enable implementation of self-care interventions with family support.

### Sample size

Sample size was estimated in the WinPepi 11.1 software suite, using readmission or emergency department visit within 90 days of randomization as the endpoint [16]. Considering a 20% relative reduction in readmissions or emergency department visits in the intervention group, a significance level of 0.05, a statistical power of 80%, and an attrition rate of 20%, the sample size was set at 270 patients (135 in each group).

### Study protocol

Participants randomized to the CG shall have their diuretic dose adjusted at the physician’s discretion. These patients will not receive telemonitoring, but will return for 30-day clinical reassessment.

Participants randomized to the IG shall receive telemonitoring with the AAD for dose adjustment and CCS for assessment of signs and symptoms of decompensation and body weight fluctuations. Telephone calls shall take place once weekly for a total of four calls during the 1-month intervention period. First, the calling nurse shall enquire as to the presence of signs and symptoms of deterioration (paroxysmal nocturnal dyspnea, orthopnea, fatigue during activities of daily living (for functional class assessment), lower extremity edema, vomiting or diarrhea, dizziness or syncope, and body weight fluctuations) during the preceding week. Depending on the signs and symptoms reported, the patient shall receive guidance according to one of the following algorithm pathways: weight remained within the expected range (change in body weight <1 kg), weight loss was greater than expected (weight loss ≥1 kg), or weight gain was greater than expected (weight gain ≥1 kg). Guidance shall be provided in accordance with the patient’s individual needs regarding adherence to pharmacological and nonpharmacological management. A face-to-face reassessment shall be conducted at 30 days by a nurse blinded to group allocation. At each telephone contact/reassessment, the AAD shall be administered again to guide diuretic dose adjustment.

Data on clinical and sociodemographic parameters shall be collected from all participants in both groups, with the CCS used to assess clinical condition.

During clinic visits, all patients are provided routine guidance on HF, including information on pharmacological management (regular use of prescribed medications and their effects) and nonpharmacological management (weight management, fluid and sodium restriction, physical activity), and any questions they may have are answered.

At the 30-day visit, all patients shall undergo reassessment by means of the CCS and recollection of data on all variables of interest.

**Figure 1:**
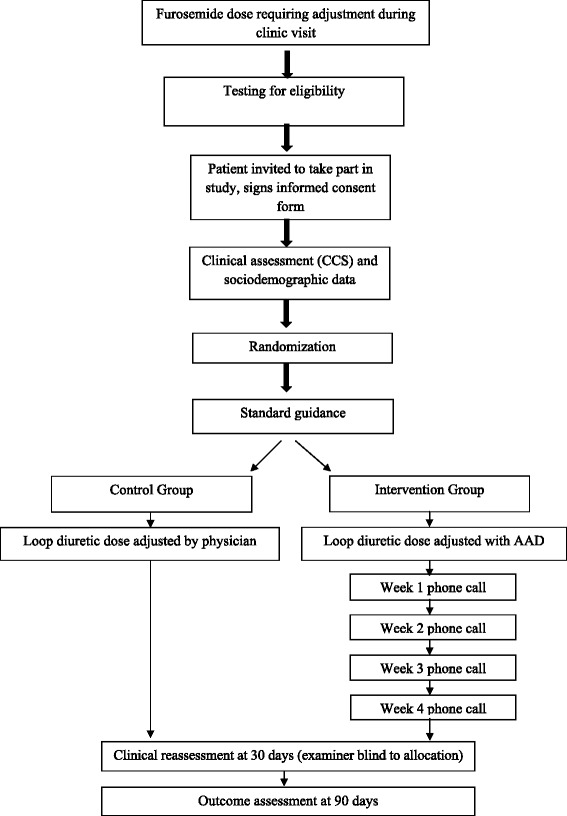
**Flowchart of study participation and interventions.**

At 90 days, the study endpoint (readmission or emergency department visit, change in CCS, deterioration in NYHA functional class, and HF-related mortality) shall be assessed by means of a chart review, consisting of an analysis of patients’ records. Patients who sought care at an outside facility shall be asked to provide their progress notes or discharge summary. Chart-based endpoints are expected to be confirmed by telephone contact with participants in both groups (Figure 1).

### Randomization

During a physician- or nurse-led clinic visit in which a need for adjustment of loop diuretic (furosemide) doses is identified, the patient shall be tested for trial eligibility by the investigator. Patients who meet the eligibility criteria shall be invited to take part in the study, and those who accept will provide written informed consent. Clinical assessment and sociodemographic and clinical data collection shall take place before randomization. The investigator shall then report to the team member (not involved in the trial) in charge of the randomization list. Specific software shall be used for participant allocation into IG and CG by sequential random number generation.

### Sociodemographic and clinical variables

A structured questionnaire shall be administered to all trial participants for collection of data on sociodemographic and clinical parameters (age, sex, educational attainment, income, current prescriptions, comorbidities, smoking, alcohol intake, etiology of HF, duration of HF, prior admissions, echocardiographic findings, and serum sodium, potassium, urea, and creatinine levels). Weight, vital signs, presence of crackles, third heart sound, jugular venous distension, peripheral edema, history of orthopnea, hepatojugular reflux, hepatomegaly, and NYHA functional classification shall be assessed during the history and physical examination. Patients shall be instructed to measure their body weight daily, in the morning, after urination, while lightly clothed and before their first meal of the day. Patients will be required to use the same scale throughout the study period and calibrate (tare) it with 1 kg before body weight monitoring [17].

### Endpoints

The endpoints include hospital readmission and/or emergency department visits due to HF decompensation within 90 days, two-point change in CCS, and deterioration of NYHA functional class. The secondary outcome shall be a composite of the aforementioned primary endpoints and 90-day HF-related mortality.

### Independent/exposure variables and confounding

In both groups, the main exposure variable shall comprise pharmacological management. The impact of each management on the trial endpoints shall be controlled for confounding variables: concomitant medications or co-intervention at an outside facility or by another provider.

### Statistical analysis

Continuous variables shall be expressed as means and standard deviations if normally distributed or medians and quartiles otherwise. Categorical variables shall be expressed as absolute and relative frequencies. Depending on the distribution of variables, baseline characteristics and the effect of the trial intervention shall be compared by means of Student’s *t-*test or the Mann-Whitney *U* test (for continuous variables) or Pearson’s chi-square test (for categorical variables). A *P* value <0.05 (two-tailed) shall be considered statistically significant. Intention-to-treat analysis shall be employed for all patients who meet a trial endpoint before 90 days. All statistical analyses shall be carried out in Statistical Package for the Social Sciences (SPSS) 18.0.

## Discussion

Patients in the intervention group (IG) and control group (CG) undergo routine assessment during physician or nurse visits by means of the CCS. This instrument tests for and scores the presence of crackles, elevated central venous pressure, jugular venous distension, third heart sound, orthopnea, hepatojugular reflux, and peripheral edema. It also assesses functional class by means of the New York Heart Association (NYHA) Functional Classification scale. Total scores range from 1 to 22. Scores ≥5 were found to be associated with congestion in a study of the clinical applicability of the AAD [14].

Data are also collected on weight, height, history of present illness, past medical history, comorbidities, echocardiographic findings, current prescriptions, sex, age, skin color, educational attainment, and household income. Laboratory parameters (serum sodium, potassium, urea, and creatinine levels) shall be recorded at randomization. If diuretic dose adjustments are required on two consecutive visits, a further battery of these tests shall be ordered.

Patients shall be instructed to return at 30 days for a face-to-face assessment visit.

## Trial status

The trial is currently in the patient recruitment stage. Thus far, 66 patients have been enrolled, 58 of whom have completed the trial.

## Abbreviations

AAD: Algoritmo de ajuste de diurético
CCS: Clinical congestion score
CG: Control group
DTA: Diuretic treatment algorithm
HCPA: Hospital de Clínicas de Porto Alegre
HF: Heart failure
IG: Intervention group
NYHA: New York heart association
PROBE: Prospective randomized open blinded endpoint
RCT: Randomized clinical trial
SPSS: Statistical package for the social sciences
U.S.: United States
